# Management of Humeral and Glenoid Bone Loss in Recurrent Glenohumeral Instability

**DOI:** 10.1155/2014/640952

**Published:** 2014-07-17

**Authors:** Randy Mascarenhas, Jamie Rusen, Bryan M. Saltzman, Jeff Leiter, Jaskarndip Chahal, Anthony A. Romeo, Peter MacDonald

**Affiliations:** ^1^Department of Orthopedic Surgery, Rush University Medical Center, Suite 300, 1611 West Harrison Street, Chicago, IL 60612, USA; ^2^Sunnybrook Hospital, University of Toronto, Room 508-A, 149 College Street, Toronto, ON, Canada M5T 1P5; ^3^Pan Am Clinic, 75 Poseidon Bay, Winnipeg, MB, Canada R3M 3E4; ^4^Toronto Western Hospital and Women's College Hospital, University of Toronto, 76 Grenville Street, Toronto, ON, Canada M5S 1B1; ^5^Department of Orthopaedic Surgery, University of Manitoba, 66 Chancellors Cir, Winnipeg, MB, Canada R3T 2N2

## Abstract

Recurrent shoulder instability and resultant glenoid and humeral head bone loss are not infrequently encountered in the population today, specifically in young, athletic patients. This review on the management of bone loss in recurrent glenohumeral instability discusses the relevant shoulder anatomy that provides stability to the shoulder joint, relevant history and physical examination findings pertinent to recurrent shoulder instability, and the proper radiological imaging choices in its workup. Operative treatments that can be used to treat both glenoid and humeral head bone loss are outlined. These include coracoid transfer procedures and allograft/autograft reconstruction at the glenoid, as well as humeral head disimpaction/humeroplasty, remplissage, humeral osseous allograft reconstruction, rotational osteotomy, partial humeral head arthroplasty, and hemiarthroplasty on the humeral side. Clinical outcomes studies reporting general results of these techniques are highlighted.

## 1. Introduction

Glenohumeral instability is a common orthopaedic problem that affects a significant number of young, active patients, with anterior instability having a reported prevalence as high as 2% [1]. In symptomatic recurrent glenohumeral instability, advanced imaging techniques are strongly recommended before proceeding to surgery in order to quantify glenohumeral bone loss, including defect size and location [2]. While nonoperative treatment options are available, surgical treatment is often the gold-standard of the therapeutic options for both glenoid and humeral head bone loss when significant bony defects exist [3]. The purpose of this review is to provide a brief overview of the anatomy of the glenohumeral joint relevant to its stability (and instability) and to illustrate the pertinent history and physical examination findings in patients with bone loss and recurrent shoulder instability. Imaging options in the workup and management of this patient population are discussed and both nonoperative and operative treatment options are described, including surgical treatment options for bone loss at both the glenoid rim and the humeral head. Finally, numerous outcome studies are discussed in the evaluation of the efficacy of these relevant surgical procedures.

## 2. Relevant Anatomy to Glenohumeral Joint Stability

The majority of the stability of the glenohumeral joint is achieved by the surrounding musculature and the extracapsular ligaments. The four muscles that comprise the rotator cuff (supraspinatus, infraspinatus, subscapularis, and teres minor) as well as the long head of biceps provide dynamic stability for the glenohumeral joint. The rotator cuff muscles provide support to the posterior, superior, and anterior aspect of the glenohumeral joint. The tendon of the long head of the biceps brachii along with the supraspinatus contributes to the prevention of superior translation of the humerus from the glenoid cavity of the scapula. The lack of musculature and the redundant capsule in the inferior aspect of the glenohumeral joint are the main contributors to the anterior/inferior instability of the shoulder joint.

The stability of the glenohumeral joint varies throughout the arc of motion and the contact between the articular surfaces reaches a maximum of 30% at a given range. When the humerus is in a hanging position, the muscles and ligaments are relaxed and joint stability is a result of intra-articular pressure [4]. In the midrange of motion, concavity compression contributes to the stability of the glenohumeral joint [5, 6], but pressure decreases as contact between the glenoid and humeral head increases [7]. The glenoid labrum deepens the shoulder cavity by up to 50% which increases concavity compression and, ultimately, joint stability. Despite this, the glenohumeral joint is least stable in the anterior/posterior direction, and at the midrange of abduction, yet if the labrum is intact. The average depth of the glenoid in the anterior/posterior direction is 2.5 mm compared to 9 mm in the superior/inferior direction [8], which explains, in part, the reason for minimum stability in the anterior/posterior direction. In addition, glenohumeral ligaments are slack in the midranges of motion [9]. Since the glenohumeral joint is least stable in the anterior/posterior direction, and the majority of dislocations occur when the humeral head translates anteriorly on glenoid, most glenoid defects occur at an area from the 3 o'clock position extending inferiorly to the 6 o'clock position [10].

The rotator cuff muscles not only compress the humeral head into the glenoid cavity but also cotension the ligaments of the shoulder. The subscapularis cotensions the inferior glenohumeral ligament complex (IGHLC) [9] which restricts the shoulder joint from reaching the endpoint of ligament function. The interval between the subscapularis and supraspinatus muscles is known at the rotator interval (RI). The RI contains the coracohumeral ligament, the superior glenohumeral ligament, and the joint capsule. A deficient RI decreases stability in the inferior directions [9]. The tendon of the long head of biceps is intra-articular and contributes to superior/inferior and anterior/posterior stability of the shoulder joint. In addition, the role of the long head of biceps tendon as a depressor of the humeral head is confirmed by the presence of superior migration of the humeral head following rupture of the tendon [11, 12]. It has also been demonstrated that the long head of biceps contributes to anterior and posterior stability during internal and external rotation of the humeral head, respectively [13].

## 3. History

Patients will often report a high-energy injury as an inciting event, especially with the arm in abduction and external rotation at the time of injury. Most will have recurrent instability and multiple atraumatic subluxation/dislocation events warranting further investigation into osseous deficiency in the unstable shoulder. It is important to note that pain may the chief presenting complaint, as the patient may not be aware that their symptoms may be secondary to recurrent subluxation of the shoulder [14]. Mechanical symptoms such as catching and/or locking can be secondary to engaging osseous defects on the humeral head and glenoid. Care should also be taken to ask questions regarding rotator cuff function in older patients that present with shoulder instability as their chief complaint. Patients may have redislocated following previous arthroscopic shoulder stabilization procedures as well and this is important to note.

## 4. Physical Exam

The affected shoulder in question should be compared to the contralateral side for the duration of the physical exam. Neurovascular status of the limb should be documented with special attention paid to axillary nerve function, while inspection should focus on any signs of deformity and muscle atrophy/wasting. Active and passive shoulder range of motion and strength of the rotator cuff muscles should be assessed. Special testing to delineate the direction and degree of shoulder instability should include apprehension and relocation [15] testing. Apprehension at small degrees of abduction and external rotation suggests glenoid bone loss, as does the ability to translate the humeral head over the glenoid rim via load-and-shift testing. The instability exam should be completed with Gagey hyperabduction testing [16], where a substantial increase in abduction on the affected side can be indicative of injury to the inferior glenohumeral ligament complex. Assessment for a sulcus sign (inferior instability) [17] and a posterior jerk test (posterior instability) [18] are also important.

## 5. Imaging

Imaging of the patient with bone loss in chronic shoulder instability should always begin with plain radiography. A trauma series consisting of anteroposterior, scapular lateral, and axillary views is recommended. Ensuring all three views are present minimizes the chances of missing a dislocation and allows for assessment of bony architecture of the glenoid and humeral head while demonstrating their positional relationship relative to one another. Axillary views are imperative in confirming glenohumeral joint congruency and can also demonstrate the presence of humeral head impression fractures, provide an assessment of glenoid erosion or fracture, and occasionally identify subchondral glenoid neck sclerosis. All of these may be indicative of chronic dislocation. Other radiographic views that may be helpful include an anteroposterior radiograph with the arm in internal rotation [19], an apical oblique view with the beam angled towards the glenoid face as described by Garth and colleagues [20], and a Stryker notch view obtained with the patients arm on top of the head and the beam centered over the coracoid process directed 10 degrees cephalad. All views can provide further ability to diagnose and quantify humeral head impression fractures. Both Strauss [21] and Danzig and colleagues [22] commented that the Stryker notch view is the most effective in demonstrating this lesion.

**Figure 1:**
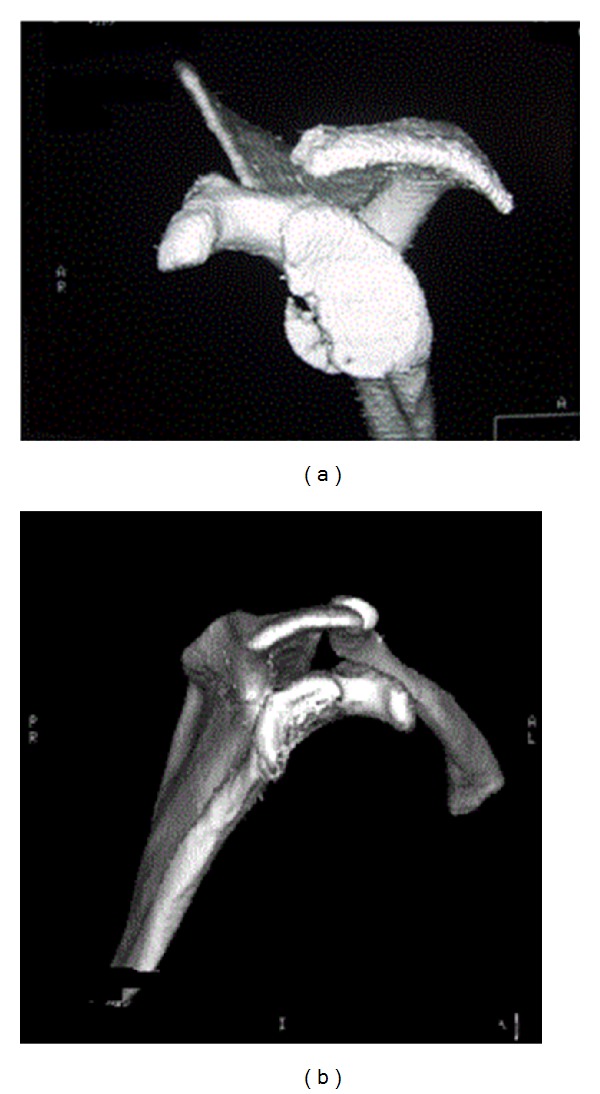
3-dimensional CT scans demonstrating (a) fragmentary bone loss and (b) attritional bone loss of the glenoid.

Modern computed tomography (CT) scanning allows for precise evaluation of the glenohumeral bony architecture in multiple planes. Multiplanar reformatting and three-dimensional reconstructions with digital subtraction techniques allow for a thorough assessment of location and size of bone loss on both the glenoid and humeral sides. Inferior glenoid bone loss can be appreciated as a percentage of its normal area when looking at sagittal imaging. A best-fit circle is used to approximate normal inferior glenoid surface area and observed bone loss can be calculated from this measurement [10, 23] (Figure 1). Typically, glenoid bone loss will lead to an “inverted pear” appearance. A study by Lo and colleagues [24] revealed that the identification of an inverted pear glenoid correlates with a minimum 25% to 27% loss of the bony width of the inferior glenoid.

**Figure 2:**
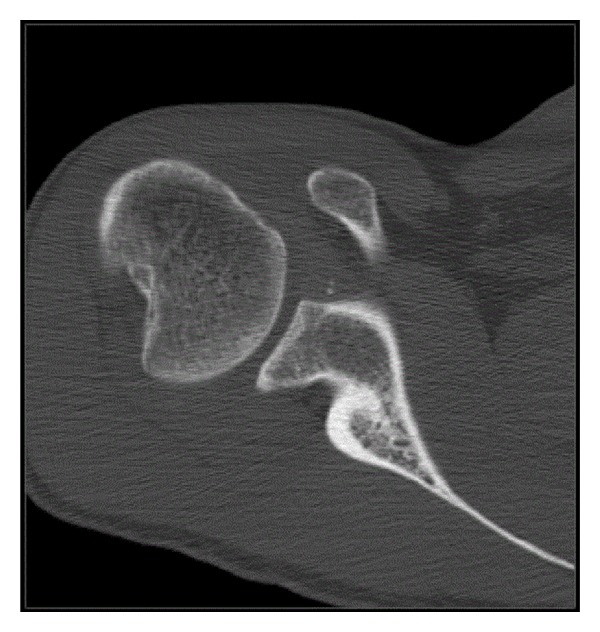
CT scan which demonstrates a large Hill-Sachs lesion.

Hill-Sachs lesions, especially those that are subtle, can also be evaluated on CT studies (Figure 2). Armitage and colleagues reported that Hill-Sachs lesions exist from 0 to 24 mm from the top of the humeral head, oriented from 6:46 cephalad to 8:56 caudal on a clock face with 12:00 defined as the intertubercular sulcus [25, 26]. The overall degree of humeral bone loss can be expressed as a percentage of articular involvement by measuring the area of impaction and dividing it by the total arc of the articular surface [27]. Treatment decisions can be made based on these measurements. Another emerging modality to image for Hill-Sachs lesions is ultrasound [28], which is readily available and avoids excessive radiation. It is limited however by interpreter skill and difficulty in discerning size and orientation of the lesion.

Magnetic resonance imaging (MRI) with or without arthrography is frequently used to evaluate the chronically unstable shoulder. In addition to providing useful information about soft tissue anatomy including the glenoid labrum, chondral surfaces, glenohumeral capsuloligamentous structures, and the rotator cuff, MRI can also demonstrate bone loss.

## 6. Treatment

Treatment of the chronically unstable shoulder should include a consideration of general patient health and function as well as an evaluation of the specific nature of the shoulder pathology present. Patient factors to consider include the presence of any significant medical comorbidities or neurological lesions, an assessment of overall functional demands, and the degree of expected patient compliance. Factors to assess with regard to the specific shoulder pathology include the chronicity of instability, the functional limitation resulting from the instability, quantification and qualification of glenoid and humeral-sided bone loss, and an evaluation of the articular cartilage in the glenohumeral joint.

### 6.1. Nonoperative Treatment

Nonoperative treatment of shoulder instability in the setting of glenoid or humeral bone loss is generally reserved for patients with significant medical comorbidities in which surgery carries unacceptably high risk, those who have low functional demands, and those who demonstrate poor compliance to postoperative rehabilitation protocols. Two particularly important subsets of patients to identify are those patients with a history of seizures or voluntary dislocations in which traditional operative intervention carries a high risk of failure.

If nonoperative treatment is selected, specific attention should be paid to a supervised rehabilitation program that emphasizes graduated range of motion exercises dependant on the amount of bone loss present. Positions that risk dislocation should be identified and avoided, as should forceful stretching. Strengthening of the rotator cuff, deltoid, and scapulothoracic stabilizers acts to improve overall shoulder function and minimize risk of future dislocation. Attempts should be made to avoid deconditioning of the shoulder musculature at all costs.

### 6.2. Operative Intervention

Operative management of recurrent shoulder instability in the setting of bone loss exists as the treatment of choice to minimize risk of future dislocation and best restore function. Goals of treatment in this situation rely on addressing both the soft tissue and bony pathology that are causative of the recurrent instability. A thorough preoperative workup consisting of appropriate history, physical exam, and imaging must be completed prior to a discussion of surgical options. Risks and benefits of each procedure must be thoroughly explained to the operative candidate, with special attention paid to the increased risk that revision surgery holds due to potentially altered anatomy and scar tissue.

**Figure 3:**
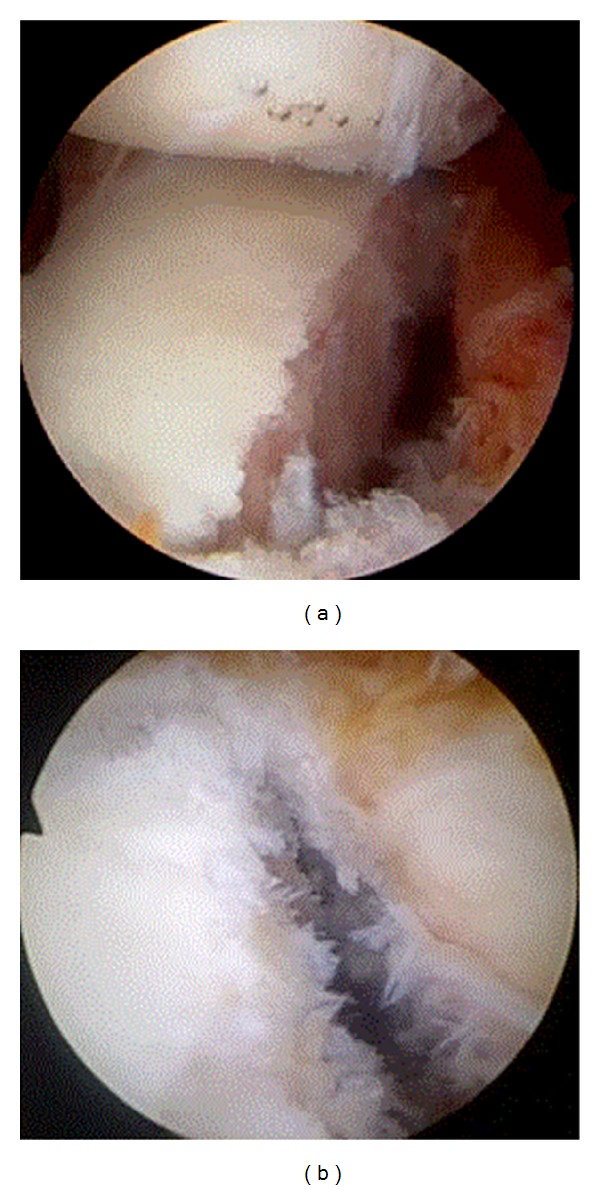
Arthroscopic views of the glenoid demonstrating (a) acute and (b) chronic glenoid bone defects in patients with chronic shoulder instability.

Quantification of the extent of bone loss has been suggested to guide operative treatment (Figure 3). Chen and colleagues [27] recommended the evaluation of the amount of humeral bone loss as a percentage of the articular surface on axillary or axial CT scan. This quantification of bone loss can be used to guide treatment toward either a soft tissue procedure alone or one of or a combination of five main types of operative procedures used with increasing bone loss: (1) humeral head disimpaction, (2) osseous/soft tissue transfer procedures, (3) osseous allograft reconstruction, (4) rotational osteotomy of the proximal humerus, and (5) partial or total humeral head arthroplasty. Similarly, Kaar and colleagues [29] found that glenohumeral stability decreased in abduction and external rotation with defects greater than 5/8 of the radius of the humeral head, and defects greater than 7/8 of the radius of the humeral head caused loss of stability in neutral abduction and external rotation. The authors thus advised reconstruction of glenohumeral defects of this size. In terms of the glenoid, Piasecki and colleagues [30] have recommended coracoid transfer or bone grafting to the glenoid be considered for defects measuring 15–25% of the total surface area, with these procedures being imperative for glenoid bone loss greater than 25%. Arthroscopic techniques can be used when bone loss is less than 15%, but attempts should be made to incorporate any bony fragments into the repair. A systematic review from Longo et al. [3] suggests that although the principle of identifying and treating glenoid and humeral bone defects in patients with traumatic anterior glenohumeral instability is acknowledged, there is a relative paucity of studies to allow definitive conclusions on the exact bone loss percentages which will increase the risk of redislocation. The authors reported in their review an overall cohort of 1817 shoulders in 1801 patients with glenoid bony defect, humeral bony defect, or both and calculated an overall redislocation rate of 6.5% (117 of 1816 shoulders), including 13.3% (30 of 225) of shoulders with humeral head bony defect, 7.2% (40 of 553) of shoulders with glenoid bony defect, and 6.3% (63 of 1009) shoulders with defects in both. These values are clinically relevant, however, when weighing the option of surgical intervention.

### 6.3. Humeral Head Disimpaction/Humeroplasty

Kazel and colleagues published on a technique aimed at restoring normal humeral head anatomy rather than simply preventing lesion engagement [31]. The procedure involves the creation of a cortical window in the mid greater tuberosity just lateral to the bicipital groove and proximal to the location of the axillary nerve. A bone tamp is inserted retrograde and a mallet is used to elevate the impacted column of bone until anatomic reduction is obtained as confirmed by direct visualization and fluoroscopy. Re and colleagues [32] published on a variation of this technique using an anterior cruciate ligament (ACL) drill guide to localize the lesion, elevated it with retrograde bone tamping, and filled the defect with cancellous bone graft [25]. Cortical screws can be inserted perpendicularly to support the correction. Little has been published in the literature with regard to indications and outcomes of this technique, but it has been suggested that disimpaction grafting is the best indicated for defects that are less than 3 to 4 weeks old and involve <40% of the articular surface. Performing this procedure in younger patients with adequate bone stock to support the articular repair can also increase the success rate [33].

### 6.4. Osseous/Soft Tissue Transfer Procedures

**Figure 4:**
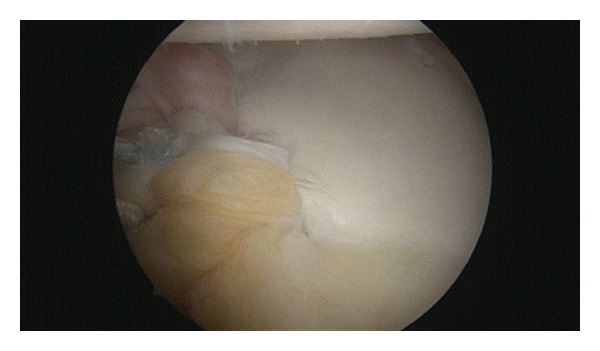
Arthroscopic view of the shoulder demonstrating the remplissage procedure.

In an attempt to address larger defects and prevent them from engaging with the glenoid, several techniques have been described to fill the osseous defect with various bony or soft tissue transfers. In the setting of chronic anterior instability, transfer of the infraspinatus tendon with or without the greater tuberosity has been used to successfully fill defects smaller than 40% of the articular surface. Initially described by Weber, the procedure combines a standard deltopectoral and separate posterior approach. Patients are positioned in either the lateral decubitus or beach chair positions in order to gain access to both the anterior and posterior shoulders. For defects measuring 20–25% of the articular surface, an infraspinatus tendon transfer can be utilized in isolation. The posterior deltoid is identified and split to reveal the infraspinatus tendon which is dissected off its attachment on the greater tuberosity. The tendon is mobilized and sutured into the defect over the lateral humeral cortex. For slightly larger defects measuring 25–40% of the articular surface, the greater tuberosity can be osteotomized and secured into the defect with two fully threaded cancellous screws after appropriate debridement of bony surfaces [33, 34]. Purchase and colleagues [35] recently described the transfer of the infraspinatus tendon and posterior capsule into the defect using an arthroscopic only technique. This remplissage procedure can be performed in both acute and chronic settings and in conjunction with anterior stabilization procedures but does not address the anatomical defect directly and can restrict motion [25, 35] (Figure 4). Following preparation of the glenoid neck and labrum for Bankart repair, visualization of the bony defect is done through the anterosuperior portal. Once the size of the defect is evaluated, its surface is prepared using a burr set on reverse. Superior and inferior suture anchors are place through a posterior or posterior accessory portal to fix the infraspinatus and posterior capsule into the defect.

McLaughlin [36] described a similar procedure for addressing the anteromedial sided humeral bone loss seen with chronic posterior instability by transferring the subscapularis tendon into the defect. Hawkins and colleagues [37] later published on the addition of a lesser tuberosity transfer to increase the stability of the repair in larger defects (40% articular surface) [33, 36]. A standard deltopectoral approach is used and the subscapularis tendon is identified at its insertion on the lesser tuberosity. Drill holes are made in a transosseous fashion through the lesser tuberosity and into the defect after appropriate debridement to bleeding cancellous bone, and the tendon is then transferred and secured in place into the defect using nonabsorbable suture. When a lesser tuberosity transfer is indicated, an osteotomy is made at its base just medial to the bicipital groove and the tuberosity is transferred into the defect and secured in place using 2 cancellous screws with the subscapularis tendon sutured overtop to the medial edge of the articular surface. This prevents the glenoid margin from falling into the defect.

### 6.5. Osseous Allograft Reconstruction

**Figure 5:**
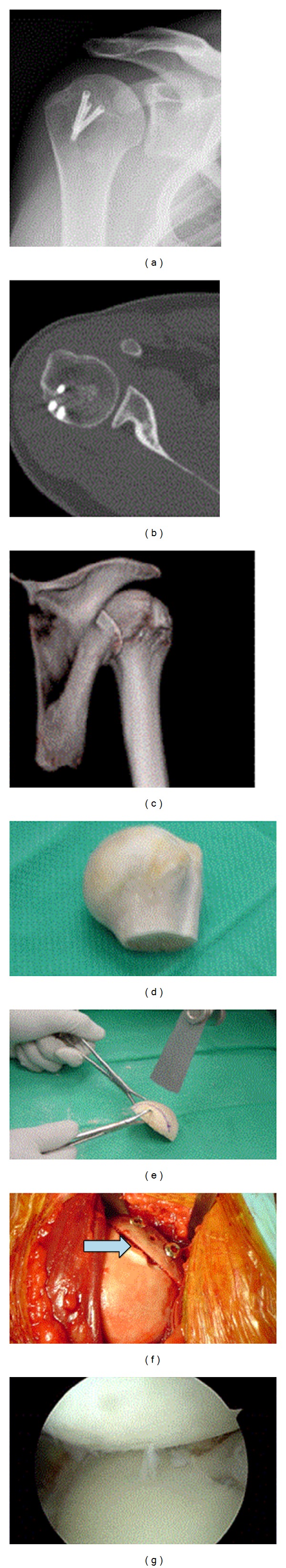
Postoperative (a) radiographic and (b)-(c) CT images following osteochondral allograft transplantation to the humeral head for a large Hill-Sachs lesion. Intraoperative pictures showcase the procured humeral head allograft (d), sizing of the graft (e), and in situ graft fixation on the humeral head (f). (g) Arthroscopic evaluation of the incorporated graft at second look arthroscopy.

Osseous allograft reconstruction exists as a solution to address moderate to large humeral-sided defects (>40% articular surface) in younger patients (Figure 5). Several authors have published on the role of this procedure in the setting of both chronic anterior instability [38] and locked posterior dislocations [39, 40] with associated bone loss. This procedure attempts to fill larger defects with both a structural and osteoconductive material in an attempt to avoid prosthetic replacement. Specific indications mainly restrict this procedure to younger patients with larger sized defects that do not have a significant degree of osteopenia or degenerative joint disease [25, 41]. After an appropriate preoperative workup that includes a CT scan to delineate humeral head bony architecture and the characteristics of the lesion, a sized matched fresh-frozen humeral or femoral head is obtained and used to graft into the identified defect. This is achieved with a standard deltopectoral approach to identify the lesion and using an oscillating saw to convert the impacted defect into a wedge of exposed metaphyseal cancellous bone. The dimensions of the graft area are measured and a sized matched allograft measuring 2 mm wider than the actual defect is impacted and secured with two cancellous screws placed through the anterolateral humeral cortex [33]. This decreases the risk of hardware prominence seen when countersunk cancellous screws are placed directly into the defect itself and the graft settles or resorbs [25].

### 6.6. Humeral Rotational Osteotomy

Rotational osteotomy of the proximal humerus is also an option that has been described to deal with large humeral head defects in younger patients to delay the need for prosthetic replacement. First described by Weber in 1969 [34], this procedure attempts to prevent an engaging Hill-Sachs lesion from contacting the glenoid and contributing to recurrent instability by allowing external rotation to be maintained through the osteotomy site. Furthermore, by utilizing rigid anatomic fixation, early rehabilitation is permitted minimizing the risk of stiffness and deconditioning of the surrounding shoulder musculature [25, 27]. Before commencing with the operative procedure, a detailed physical exam must be performed making sure to document the exact degree of range of motion that causes dislocation, specifically external rotation. Once range of motion is documented, a standard deltopectoral approach is utilized to expose the proximal humerus and an oscillating saw is then used to complete a transverse osteotomy through the surgical neck. The humeral shaft is rotated externally to 5–10 degrees more than the position of instability measured on physical exam and the osteotomy is then secured using a rigid fixation implant such as a blade plate. Imbrication of the anterior capsule and subscapularis tendon is then done in conjunction with the bony procedure.

### 6.7. Partial Humeral Head Arthroplasty

Partial resurfacing of large humeral head impression fractures with a cobalt-chrome articular component is an emerging technique in younger patients which may decrease the risks seen with other osseous procedures. While previously described in the literature for glenohumeral osteoarthritis, avascular necrosis, and rheumatoid arthritis [42], several authors [43, 44] have since published on the use of these implants in the setting of chronic instability. The advantages proposed in using these implants include absence of donor site morbidity compared with autograft, shorter operative time, no associated graft resorption and hardware removal, and lack of disease transmission [25, 43]. On the other hand, disadvantages include potentially inadequate fixation of the implant to the humeral head, a mismatch between the implant and defect geometry that may require further reaming and resurfacing of unaffected humeral cartilage, and an inability to accurately align the surface of the prosthesis with the adjacent articular surface [25, 44]. The operation is performed through a standard deltopectoral approach to adequately expose the humeral head defect and evaluate the glenoid for bone loss. Using preoperative and intraoperative measurements, an implant of appropriate size is selected to match the patient's particular anatomic defect and secured into place. Stability should be tested intraoperatively to ensure correction of the instability prior to completing the operation. Any residual instability may indicate the necessity to perform an additional bony or soft tissue procedure.

### 6.8. Complete Humeral Head Resurfacing/Hemiarthroplasty

Complete humeral head resurfacing or hemiarthroplasty has been described as being indicated in older patients with impression fractures greater than 40% of the articular surface and younger patients with chronic defects and significant articular cartilage degeneration [33]. Due to the limited lifespan of these implants, evaluation of patient suitability for any of the aforementioned procedures is necessary prior to committing to humeral head resurfacing or hemiarthroplasty. Furthermore, with evidence supporting better outcomes of primary total shoulder arthroplasty over isolated hemiarthroplasty for glenohumeral osteoarthritis [45, 46], a thorough evaluation of glenoid sided pathology including articular cartilage degeneration and bone loss is imperative. The procedure is performed through a standard deltopectoral approach to expose the proximal humerus. In the setting of chronic anterior instability it has been suggested that increasing the amount of retroversion by 10–15 degrees may increase stability. Accordingly, decreasing the retroversion by 10–15 degrees in the setting of posterior instability may further stabilize the glenohumeral joint [33, 37]. A thorough assessment of the glenoid and final testing of stability needs to be performed prior to completing the operation. Any residual instability or glenoid articular wear needs to be addressed through either glenoid-sided bone grafting, total shoulder arthroplasty, or soft tissue imbrication [33].

### 6.9. Arthroscopic and Open Capsulolabral Reconstruction

**Figure 6:**
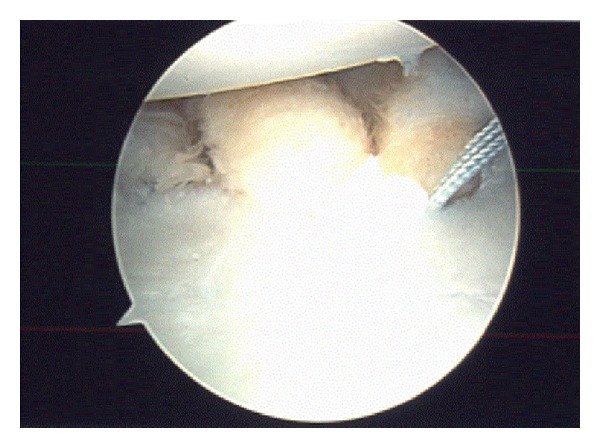
Arthroscopic image of a posteroinferior labral repair through a 7 o'clock portal as an adjunct to anterior stabilization in a patient with recurrent shoulder instability.

Arthroscopic techniques may be used for osseous defects that measure less than 25% of the glenoid. This may be done using various suture anchors placed on the anterior glenoid neck with the most important factors being restoring appropriate capsulolabral tension and trying to incorporate any bony fragments in the reconstruction. Open capsulolabral repair may be performed in the same circumstances and may be easier to perform in revision instability cases. This technique is performed through a standard deltopectoral incision with the labrum dissected off the glenoid neck to allow osseous preparation down to a bleeding bone surface with a rasp. Suture anchors are then placed on the anterior glenoid neck to facilitate repair of the labrum with imbrication of the inferior aspect of the glenohumeral capsule into the labral repair (Figure 6).

### 6.10. Coracoid Transfer Procedures

**Figure 7:**
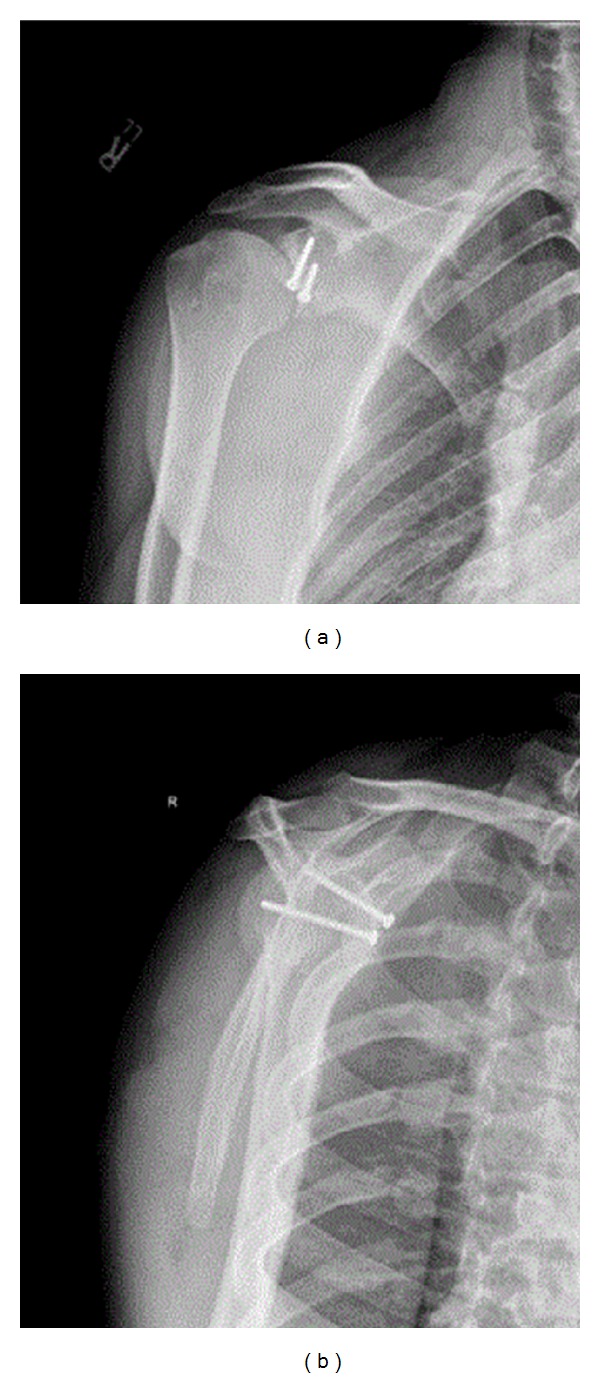
Two views of the right shoulder following an open Latarjet stabilization procedure for recurrent right shoulder instability.

**Figure 8:**
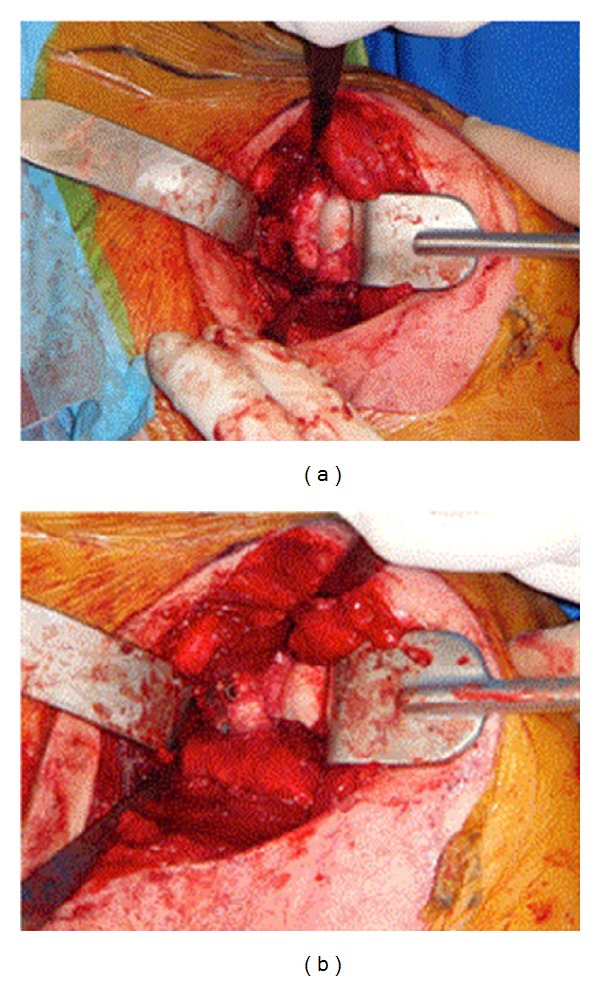
Intraoperative photographs demonstrating the Latarjet procedure through subscapularis split.

For patients with significant anteroinferior glenoid bone loss (>25%), various coracoid transfer procedures have been described. Helfet described the Bristow procedure, where the tip of the coracoid was osteotomized and transferred to the glenoid neck just medial to the rim [47]. This procedure was nonanatomic and largely relied on the glenohumeral restraint offered by the sling effect of the coracobrachialis with the arm in abduction and external rotation. The Latarjet procedure, on the other hand, requires removal of a much larger portion of the coracoid (2-3 cm) with transfer along its long axis to the anteroinferior glenoid neck [48] (Figures 7 and 8). This allows for the sling effect provided by the coracobrachialis but also attempts to reconstruct the osseous anatomy of the glenoid. This restoration of anatomy can deepen the glenoid cavity and restore the glenoid arc, making it more difficult for a concomitant Hill-Sachs lesion to engage the anteroinferior glenoid rim and increasing the amount of humeral head excursion required for dislocation [49]. The Latarjet coracoid transfer can also serve as reinforcement to anteroinferior capsular deficiency. Lafosse et al. [50] has recently described an arthroscopic Latarjet technique, which offers better visualization of coracoid fixation on the glenoid and thus theoretically reduces the risk of iatrogenic osteoarthritis. This benefit is offset by the immense difficulty of the procedure, even in experienced hands [51].

### 6.11. Anatomic Allograft and Autograft Reconstruction

**Figure 9:**
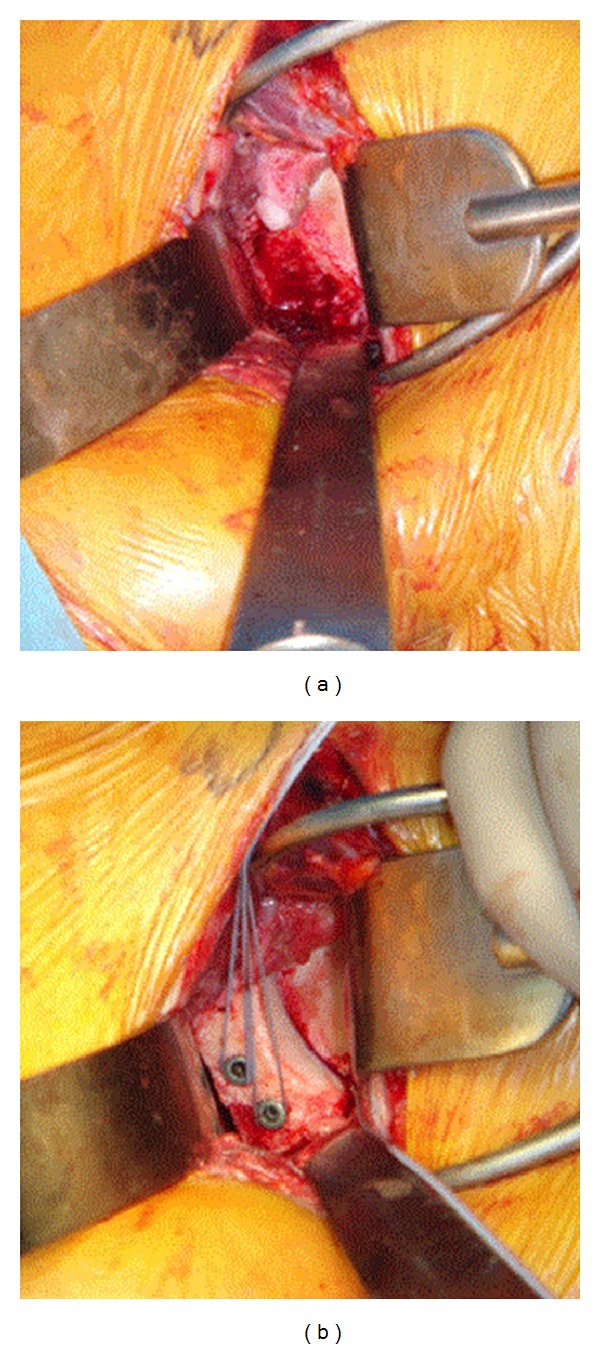
Glenoid augmentation with distal tibial osteochondral allograft.

Autogenous bone grafting procedures of the glenoid, mainly with iliac crest, are gaining increasing popularity secondary to suggestions that they may offer a more anatomical reconstruction of the glenoid and thus restoration of the natural articular arc [52]. In order to avoid the morbidity associated with iliac crest harvest, the use of various allograft sources has also been described and includes iliac crest [53], distal tibia [54], and frozen femoral head [55] (Figure 9). Regardless of the graft source, the procedure is performed through a standard deltopectoral incision with exposure and preparation of the anterior inferior glenoid with a burr. The graft is prepared to fit the glenoid defect with a saw and provisionally secured into the defect with k-wires. The graft is then secured with a lag technique using one or two 3.5 mm fully threaded cortical screws. Some techniques advocate placing the bone block after repair of the labrum and capsule (extracapsular), while others suggest placing the bone block within the capsule and subsequently repairing the remaining labrum and capsule to the extent that tissue quality allows. Although there are not sufficient outcome data to conclude which of these two techniques is superior, the former provides a soft tissue interposition between the humeral head articular surface and the bony graft, while the latter allows the humeral articular surface to sit on exposed nonarticular bone graft which may increase the risk of arthrosis. Ultimately the patient is placed in an abduction brace.

### 6.12. Postoperative Management

Postoperatively, patients should be rested in a sling to allow for healing and can slowly be mobilized making sure to avoid positions that would allow the humeral head to engage the glenoid. Passive and active-assist exercises are allowed while avoiding external rotation and abduction. After 4 to 6 weeks, patients can begin active and resisted range of motion exercises while avoiding contact sports and positions which risk dislocation. This is followed by progressive strengthening and sports/work specific activities.

## 7. Clinical Outcomes

### 7.1. Reconstruction of Glenoid Bone Loss in Recurrent Shoulder Instability

Beran et al. [56] recently conducted a systematic review in order to evaluate the literature regarding treatment of chronic glenoid bone defects to determine if one surgical glenoid reconstruction technique could be recommended over another in patients with recurrent anterior shoulder instability. After excluding studies that did not report follow-up or quantify glenoid deficiency, or performed open reduction and internal fixation/capsulolabral repair for glenoid rim fractures, there were six eligible manuscripts [53, 55, 57–60]. All six of these studies were level IV evidence (case series) with 5 being retrospective [53, 57–60] and one being prospective [55]. In total, these studies analyzed 134 shoulders. The mean age of the patients was 31.6 years; 10.5% were female and the combined mean follow-up was 55.6 months. The study by Burkhart et al. [58] looked at the open modified Latarjet technique while the remainder of the studies looked at either an open allograft bone block technique [55], an open J-graft autograft technique [57], or an open iliac crest autograft bone block technique [53, 59, 60]. All patients across the various studies were also treated with a capsulorrhaphy.

The results of the aforementioned systematic review reported a recurrent instability rate from 0 to 4.9% [56]. All studies reported >90% return to sports. In regard to functional outcomes, four of the six studies used the Rowe score (0–100) [53, 55, 57, 60]. The mean Rowe across these four studies was 90.5 (excellent). The remaining two studies reported overall Constant scores of 94 [59] and 94.4 [58]. It should be noted that there was no observed increase in motion loss in patients undergoing a Latarjet reconstruction as opposed to the more anatomic bone grafting reconstruction in the included studies [56].

In regard to the effect of glenoid reconstruction on the long-term risk of glenohumeral osteoarthritis, only two of the six studies in the review by Beran et al. [56] were evaluated for arthritis changes. Scheibel et al. [60] demonstrated that, of the ten patients in their series, two patients had grade I arthritis and one had grade II arthritis via the Samilson/Prieto classification. In the study by Auffarth et al. [57], 19 of 47 shoulders showed arthritic change at a follow-up of 72 months with 11 of these patients having preoperative evidence of arthrosis. Although not included in the systematic review due to failure to quantify the amount of glenoid deficiency, Hovelius et al. [61] have looked at the onset of glenohumeral arthropathy 15 years following 118 cases of coracoid transfer for recurrent shoulder instability. At fifteen-year prospective follow-up, 3.4% of patients had one or more recurrences of instability. In regard to arthropathy diagnosed using an AP projection of the shoulder, 34.2% of patients had mild arthropathy, 4.4% had moderate arthropathy, and 1.8% of patients had severe arthropathy. Hovelius et al. [61] also indicated that there was no association between the degree of loss of external rotation in the operative shoulder at two years and arthropathy at fifteen years. Other studies with long-term follow-up (14–20 years) have demonstrated moderate to severe arthropathy rates following a coracoid transfer ranging from 19% to 28.6% [48, 62]. Rahme et al. [63] have reported that 30% of shoulders had moderate or severe arthropathy at 22–37-year follow-up following a bone block glenoid reconstruction procedure. The superiority of one technique over another in preventing or contributing to the long-term development of arthrosis in the recurrent dislocator cannot be delineated based on the currently available literature. Based on the limited strength of the representing studies, one technique could not be recommended over another. In addition, between different bone block reconstruction techniques, there is no clear delineation of outcomes between allograft and iliac crest bone graft. Nonetheless, all of these methods appear effective in restoring and maintaining stability. All techniques are associated with the long-term development of arthrosis.

More recent patient series have indicated that the Latarjet procedure may be a superior treatment option for chronic anterior instability in high-level athletic patients with large glenoid bone defects. Cerciello et al. [64] retrospectively reviewed 28 shoulders in 26 soccer players affected by chronic anterior instability. At a mean of 85 months following the Latarjet procedure, the mean Duplay score was 89.3 and most players returned to the same high level of soccer. Only 1 player redislocated, and 93% of patients were “happy” or “very happy” with the results. A modified Latarjet procedure with a coracoid bone block was successful in achieving increased patient satisfaction and return to former activity levels in 35 patients (mean age 35 years, range 20–58) in a study by Atalar et al. [65]. The coracoid graft demonstrated osseous union in all patients at a mean 24 ± 12.2 months (range, 12–74) with no further instability or degenerative arthritis. Mean VAS scores decreased significantly, from 6.2 ± 2.4 to 1.8 ± 0.6.

### 7.2. Humeral Head Bone Loss in Recurrent Shoulder Instability

There is a paucity of literature with long-term follow-up related to the surgical reconstruction of humeral head defects in patients with recurrent shoulder instability. Armitage et al. [25] have conducted a succinct review of the outcomes to date following the current spectrum of procedures which includes humeroplasty, remplissage, osteoarticular allograft, rotational osteotomies, and partial resurfacing.

Re et al. [32] have reported the results of a humeroplasty technique in four patients with associated capsulolabral repair or Latarjet transfer. There were no recurrences at one-year follow-up. It has been suggested that humeroplasty is likely the most beneficial for an acute injury [25]. While a few cadaver studies have reported positive results from the procedure [66, 67], further clinical outcomes research is required in regard to this technique.

In regard to the arthroscopic remplissage procedure, Purchase et al. [35] reported a recurrence rate of 7% and no loss of shoulder motion following this procedure. Lynch et al. [68] reported satisfactory results in 14 of 15 patients treated with open transfer of the infraspinatus tendon for large defects of the humeral head and noted no significant complications nor limitations in rotation. No information regarding functional outcomes using shoulder-joint specific outcome measures was reported. Long-term outcomes on the treatment of traumatic anterior shoulder instability with both glenoid bone loss (grade IIIA) and significant Hill-Sachs lesions with arthroscopic remplissage were reported by Wolf and Arianjam [69]. At a mean 58 months (range, 2–10 years) after the procedure, two of 45 patients (4.4%) in this cohort had recurrent instability after athletic events, with no complications or reoperations seen in the remaining 43 patients. Excellent outcomes were obtained on Rowe scores, Constant scores, and Western Ontario Shoulder Instability Index at final follow-up. A systematic review of largely heterogeneous studies on arthroscopic “remplissage” for shoulder instability reported apparent success of the procedure from 8 articles with a total of 207 patients, reporting a mean redislocation rate of 4.2 ± 3.9% (range, 0–15%) and a mean recurrent instability rate of 3.2 ± 3.8% (range, 0–15%) [70]. Mean reductions in external rotation in adduction, external rotation in abduction, and internal rotation were reported as 5.6° (−40 to +30), 11.3° (−50 to +7), and 0.9 (−4 to 0) vertebral levels, respectively.

Miniaci and Gish [71] have reported on 18 cases with the use of osteoarticular allograft for reconstruction of humeral head defects. All patients had failed instability repairs and had humeral head defects greater than 25% of the articular surface. At final follow-up, the Western Ontario Shoulder Instability (WOSI) index had improved in all patients and the average Constant score was 78.5. More than 89% of patients returned to work and no patients had recurrent instability. Dipaola et al. [72] also reported on the results of osteoarticular allograft for the management of humeral head defects following recurrent shoulder instability. At a follow-up of 27.4 months, the average ASES and UCLA shoulder scores were 85.3 and 28.4, respectively. The average loss of external rotation was 8 degrees and no patients in their study demonstrated recurrent instability. The long-term follow-up of allograft reconstruction of humeral head segmental defects from posterior shoulder dislocation was evaluated by Martinez et al. [73]. In 6 men with defects consisting of 40% articular surface involvement, all patients returned to their previous occupation by 4 months after operation. At a mean of 122 months (range, 96–144) after the surgery, half of these patients had no complaints of pain or instability and had good results. The three patients without good results had osteoarthrosis requiring shoulder arthroplasty after 8, 8, and 10 years, respectively.

Weber et al. [74] conducted a review of 180 rotational subcapital humeral osteotomies with shortening of the subscapularis tendon and capsule for recurrent shoulder instability. The overall redislocation rate was 5.7% and the rate of nontraumatic redislocation was 1.1%. Limitation of motion of more than 10 degrees was present in only 3.9% of patients. The average loss of external rotation was less than 5 degrees without noticeable diminution of power or function in most patients. The results were good to excellent in 90% of cases as determined by the Rowe score. Plate removal was performed one to two years postoperatively in 107 of the 180 shoulders. All athletes in the series returned to previous levels of function, including 14 professionals. Kronberg and Brostrom [75] reported their five-year results of 20 derotation osteotomies performed for recurrent instability. They reported no cases of recurrent instability, infection, nonunion, or neurological sequelae. Although not statistically significant, there was an 8 degree deficit in internal rotation postoperatively.

The surgical outcomes following partial resurfacing of humeral head defects are currently limited to very small case series or case reports [43, 44]. Based on this, we cannot advocate for or against this technique at the present time.

## 8. Conclusion

Recurrent glenohumeral instability is a difficult orthopaedic problem that requires specific history and physical examination to delineate whether bony deficiency may be the root of the problem. Various imaging modalities are paramount to allow for quantification of bone loss and surgical planning, and numerous techniques exist for reconstruction of both humeral and glenoid sided defects. There are few studies comparing the various techniques, and although good to excellent results are reported in a large number of case series, more prospective cohort studies and randomized controlled trials need to be conducted in order to determine which techniques provide the best long-term outcomes in terms of stability, motion deficits, functional outcome scores, and the development of arthrosis. Smaller defects can be treated effectively with arthroscopic soft tissue procedures on both the glenoid and humeral head, while larger defects require osseous reconstruction to restore glenohumeral stability. Older patients with concomitant degenerative glenohumeral joint disease may require some form of arthroplasty to offer them relief.
